# Statistical Analysis of Safety Performance of Displaced Left-Turn Intersections: Case Studies in San Marcos, Texas

**DOI:** 10.3390/ijerph17186446

**Published:** 2020-09-04

**Authors:** Wenrui Qu, Qiao Sun, Qun Zhao, Tao Tao, Yi Qi

**Affiliations:** 1 School of Mathematics and Statistics, Qilu University of Technology (Shandong Academy of Sciences), Jinan 250353, China; quwenrui601@163.com; 2 Department of Transportation Studies, Texas Southern University, Houston, TX 77004, USA; qiao92ashin@gmail.com (Q.S.); qun.zhao@tsu.edu (Q.Z.); tao.tao@tsu.edu (T.T.)

**Keywords:** displaced left-turn design, safety performance, before-and-after study, collision diagram

## Abstract

Displaced left-turn (DLT) intersections are designed to increase the mobility of vehicles by relocating the left-turn lane (lanes) to the far-left side of the road upstream of the main signalized intersection. Since DLT is a relatively new design and very limited crash data are available, previous studies have focused mainly on the analysis of its operational performance rather than its safety performance. To fill this gap, in this study, we investigated the safety performance of two DLT intersections located in San Marcos, Texas. Crash data from 2011 to April 2018 were extracted from the TxDOT Crash Record Information System (CRIS). These crash data were analyzed using two different approaches, i.e., statistical analysis and collision diagram-based analysis. The results of this study indicated that DLT did not increase the overall crash frequencies at the studied intersections. Traffic crashes related to left turns and right turns were reduced significantly by DLT. Meanwhile, it also caused safety issues related to traffic signage, traffic signal, geometric design, and access management at DLT intersections. Thus, in the implementation of DLT intersections, traffic engineers need to carefully consider different aspects of the DLT intersection design, including access management, traffic signal coordination, and driver acceptance. As a result of these analyses, recommendations were provided for the safe implementation of the DLT design in the future.

## 1. Introduction

**Figure 1:**
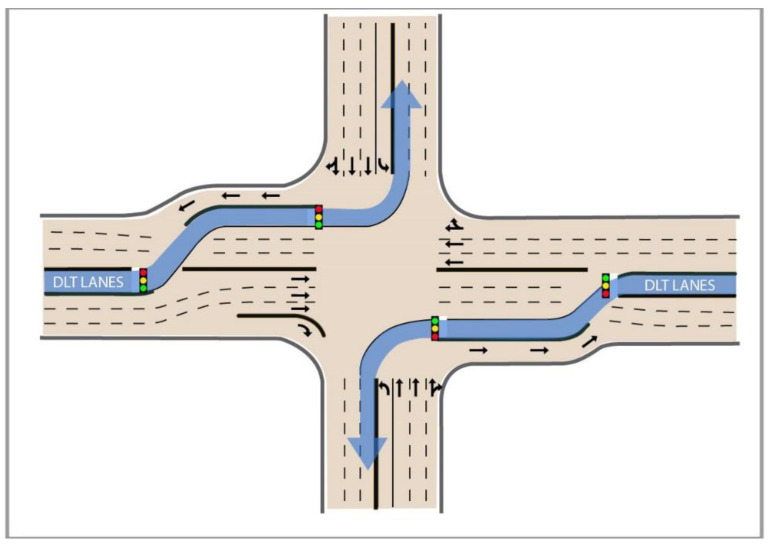
Displaced left-turn (DLT) layout (partial DLT with two crossovers).

Intersections with the displaced left-turn (DLT) design are also known as continuous flow intersections (CFIs). Such innovative intersections were designed to increase the mobility of traffic by relocating the left-turn lane (lanes) to the far-left side of the road upstream of the main signalized intersection. Figure 1 shows the design concept of DLT and its left-turn routes. In this design, protected left turns can be completed simultaneously with the through movements of other vehicles, so the traditional four-phase signal control can be replaced by simple two-phase control. As a result, the capacity and operational efficiency of intersections can be improved significantly. Although previous studies have indicated that the DLT design has the potential to improve the operation and safety of signalized intersections [1,2,3,4,5,6], its safety impact needs to be investigated further because rerouting vehicles that are going to make left turns at upstream locations could increase the risk of crashes at these locations. In addition, drivers’ acceptance of the new design also needs to be investigated.

## 2. Literature Review

DLT is being used increasingly in several states in the U.S., and some studies have been conducted to investigate the performance of this innovative design. However, previous studies have focused mainly on the operational performance, and the safety impacts of DLT have not been investigated thoroughly. Pitaksringkarn conducted a traffic simulation-based study to assess the operational performance of DLT, and the results indicated that DLT intersections had a significant reduction in average delay compared with traditional intersections [7]. A subsequent study conducted by Cheong et al. reported similar findings, with the DLT intersections outperforming conventional intersections for both the movements of through-only traffic and left-turn-only traffic in various scenarios [8].

Other researchers also have reported that DLT has successfully reduced intersection travel delays, number of stops, and queue lengths and increased intersection capacity. For example, Kim et al. found that, compared with the conventional T-intersection, the DLT intersection can result in a significant reduction of delay for through and left-turn movements [9]. Esawey and Sayed compared DLT with a similar conventional intersection under two scenarios: balanced and unbalanced traffic volume using traffic simulation-based experiments. The results showed that the DLT intersection exhibited lower delay than the conventional intersection under all balanced volume levels especially under moderate- and high-volume conditions. In addition, the capacity of the DLT intersection was higher than that of the conventional intersection by about 90% [10]. Jagannathan and Bared used the traffic simulation-based study to compare three different DLT design configurations with conventional designs. Their results revealed that the DLT intersections consistently outperformed the conventional intersection, even at low traffic volumes [11].

Some studies also compared the operational performance of DLT intersections with other innovative intersections. For example, Dhatrak compared the operational performance of DLT and parallel flow intersection (PFI) based on the maximum through and left-turn movement throughputs for three different high-volume scenarios using traffic simulation. The simulation results showed that the maximum throughput values of through movement in both designs were very close, but left-turn movement throughputs in DLT were higher than those at PFI [12].

Olarte compared the operational performance of left-turn bypass, diverging flow, and displaced left-turn intersections. The results indicated that the DLT consistently operated with less delay in almost all cases [13].

Compared to a large number of studies on the operational performance of DLT intersections, very few studies have focused on the safety impacts of such intersections. Federal Highway Administration (FHWA)’s Displaced Left-Turn Intersection Informational Guide indicated several safety outcomes of implementing the displaced left-turn design: (1) DLT has successfully reduced the conflict points by 6–12% in four-leg intersections compared to conventional intersections, (2) the DLT design can reduce the delay time and the number of stops on a major road, which could reduce the frequency of rear-end crashes, and (3) the DLT crossover layout increases the risk of wrong-way movements [14].

Louisiana Department of Transportation and Development (LaDOTD) evaluated the safety performance of a pilot DLT intersection at Airline Highway/US 61 and Siegen Lane/Sherwood Forest Boulevard in Baton Rouge, LA, by conducting a before-and-after analysis of crash data. This is a partial DLT design with two crossovers located on the 200-foot-wide Airline Highway [8]. They measured the crash data for two, two-year periods (2002–2003 and 2004–2005) before the installation of DLT, and they also measured the crash data for a 17-month period after the installation of DLT. The results showed that the numbers of accidents were reduced from 185 and 200 in 2002–2003 and 2004–2005, respectively, to 146 after the implementation of the DLT design, which represented reductions of 21% and 27%, respectively. In addition, the frequency of accidents involving significant injuries was reduced by 17%. However, LaDOTD’s Highway Safety Section recommended that at least a three-year period be used to collect post-crash data in order to conduct a proper evaluation of the safety performance of DLT intersections [15]. Therefore, additional studies will be required to acquire adequate crash data after the installation of DLT at intersections.

Park and Hesham assessed the safety impacts of DLT based on the analysis of traffic conflicts [16]. They selected two DLT intersections in Louisiana to analyze the drivers’ behaviors at these innovative intersections. Traffic videos were recorded at these intersections for two periods, i.e., immediately after the DLT was opened and one year after the DLT was opened. The traffic conflicts they identified included “improper lane change”, “diverge”, and “red light violation”. Their results indicated that drivers’ lack of familiarity with the displaced left-turn design might cause unexpected driving maneuvers.

Zlatkovic accessed the safety performance of DLT intersections by developing crash modification factors (CMFs) using the empirical Bayes (EB) methodology [17]. Eight DLT intersections along Bangerter Highway in Utah were selected to acquire the available before and after crash data and annual average daily traffic (AADT) between 2008 and 2013. Crashes that occurred within 100 feet (30.48 m) for each crossover and 250 feet (76.2 m) for the main intersection were summed to provide the total crash data for the intersection. According to EB analysis results, the crash modification factor for DLT conversion was 0.877, which indicated that the DLT design had the potential to reduce crashes.

However, Abdelrahman et al. found that DLTs could have a negative impact on intersection safety. In their study, the authors evaluated the safety performance of DLTs using two methods, a before-and-after study with a comparison group and a cross-sectional analysis. Both results showed that DLTs can significantly increase the total number of crashes as well as injury crashes and some other crash types (i.e., single vehicle, and angle). However, they have the potential to decrease non-motorized crashes and head-on crashes. In addition, the authors also found that some other factors have significant effects on the crash frequency. For example, they found that crash frequency decreases when the minor street has a low-speed limit [18].

From the literature mentioned above, it is evident that most of the previous studies focused on the operational performance of DLTs. Very few studies evaluated the safety impacts of the DLT design, and these impacts have been analyzed mainly through the statistical analysis of historical crash data. Since the statistical analysis depends on the general crash attributes contained in the crash database, some local specific risk factors related to the crash location and its surrounding environment cannot be identified. To fill this gap, in this study, two different approaches were used for an in-depth assessment of the safety performance of two DLT intersections in San Marcos, Texas. These two approaches are (1) statistical analysis of crash data and (2) collision diagram-based analysis. By comparing the safety performance before and after the implementation of DLT at the intersections, the safety impacts of the DLT were analyzed, and recommendations for future implementation of DLT were provided. The results of these studies will provide traffic engineers/designers with a better understanding of the safety impacts associated with the implementation of the DLT design, and the results also will assist them in designing, installing, and managing better DLT intersections in the future.

## 3. Methodology

### 3.1. Study Sites

Two DLT intersections were selected as study locations, i.e., Loop 82 at Interstate Highway (IH) 35 and State Highway (SH) 80 at IH 35 in San Marcos, Texas. The Loop 82 project was completed on 1 May 2014 and the SH 80 project ended on 12 October 2014. These two DLT intersections were selected due to the following criteria.

(1) The DLT intersections are in Texas. For all the crashes that occurred in Texas, the research team had full access to not only the crash data, but also the detailed police crash reports for conducting collision diagram-based analysis.
(2) The DLT intersections need to be implemented for more than 3 years. In this way, enough crash data before and after the implementation of the DLT design can be available for conducting a statistical analysis.
(3) These two DLT intersections represent the two different types of DLT design: single-leg DLT (Loop 82 at IH 35) and two-leg DLT (SH 80 at IH 35).

To analyze the safety impacts of the DLT design at these two intersections, crash data for the past eight years (January 2011–April 2018) were obtained from TxDOT’s Crash Records Information System (CRIS) for conducting the before and after studies. In addition, detailed police crash reports for these crashes were also extracted from the CRIS system.

For Loop 82 at IH 35, we studied a total of 36 months of data (from January 2011 to December 2013) before DLT was installed, a total of 4 months of data (from January 2014 to April 2014) during the construction, and a total of 48 months of data (from May 2014 to April 2018) after DLT was installed. In total, 336 crashes occurred at this intersection during the study time period.

For SH 80 at IH 35, we studied a total of 40 months of before data (from January 2011 to April 2014), a total of 5 months of data (from May 2014 to September 2014) during the construction, and 43 months of after data (from 12 October 2014 to April 2017). In total, 513 crashes occurred at this intersection during the study time period.

### 3.2. Safety Analysis Methods

For these two intersections, two different approaches were used to analyze the safety impacts of converting a conventional intersection to a DLT intersection, i.e., a statistical analysis and a collision diagram-based analysis.

#### 3.2.1. Statistical Analysis

For the statistical analysis approach, the following two methods were used. First, a direct comparison between the monthly crash frequency before and after implementing the DLT was conducted. Note that, for both study locations, crashes that occurred during the construction period were excluded from the data to eliminate crashes caused by construction. A statistical *t*-test was conducted to ensure that the difference in crash frequency was statistically significant.

Second, the empirical Bayes (EB) method was applied to account for the effects of the changes in traffic volume before and after the implementation of DLT. The basic idea is that the changes in accident frequency may be due to contributing factors other than the treatments (the implementation of DLT in this study). Therefore, in order to assess the true effects of the treatments, the expected number of accidents that occurred during the after-treatment period in the absence of treatment will be estimated and compared with the observed number of accidents that occurred during the after-treatment period. If the expected accident number is less than the actual observed number, the treatment is effective; otherwise, it is ineffective or even adversely affects the intersection safety.

The EB method combines the observed crash frequency with the predicted average crash frequency to produce a more statistically reliable measure, which is referred to as expected crash frequencies. The details about the use of the EB method for transportation safety performance analysis can be found in Highway Safety Manual [19]. To use the EB method, a safety performance function was selected for predicting the crash frequency under the assumption of no treatment, i.e., $E_{crash}^{after\_no\_treatment}$, during the after-study time period. For this purpose, an existing model for predicting the crash frequency at signalized diamond interchange ramp terminals developed by Wang et al. [20] was selected as the safety performance function (SPF). This model can be expressed mathematically as follows:

(1) $$E_{crash}=a\times VE^{b}\exp(c\times Y_{dif}+d\times AR_{dif}+eRT_{dummy}+f\times S_{terminal})$$

where $E_{crash}$ is the expected crash frequency at the signalized diamond interchange ramp terminals; VE is volume exposure, reflecting the actual effect of volume and it is determined by the AADT of two off-ramps and the AADT of the crossroads at the two ends of ramp terminals; $Y_{dif}$, $AR_{dif}$, and $eRT_{dummy}$ are variables related to the traffic signal timing; $S_{terminal}$ is the spacing between two ramp terminals in feet; *a*, *b*, *c*, *d*, *e*, and *f* are constants or the coefficients of independent variables and their values are given by Wang et al. [20].

According to Equation (1), since it is assumed that there is no treatment at this location, i.e., both the traffic control and geometric conditions stay the same at this location and the expected crash frequency during the after-study time period, $E_{crash}^{after\_no\_treatment}$, can be estimated as follows:

(2) $$E_{crash}^{after\_no\_treatment}=\frac{VE_{after}^{b}}{VE_{before}^{b}}E_{Crash}^{Before}$$

where $E_{crash}^{before}$ is the expected crash frequency during the before-study time period; $VE_{before}^{}$ and $VE_{after}^{}$ are the volume exposure during the before- and after-study time periods, respectively. According to Wang et al. [20], $VE=AADT_{Ramp1}\times AADT_{crd1}+AADT_{Ramp2}\times AADT_{crd2}$, where $AADT_{Ramp1}$ and $AADT_{Ramp2}$ represent the AADT of two off-ramps; $AADT_{crd1}$ and $AADT_{crd2}$ represent the AADT of the crossroads at the two ends of ramp terminals. According to Equation (2) and the traffic volume conditions at this location before and after the implementation of DLT, the expected crash frequency during the after-study time period under the assumption of no treatment, i.e., $E_{crash}^{after\_no\_treatment}$, can be estimated. After that, it can be compared with the observed crash frequency of the same intersection with the treatment during the after-treatment period, i.e., $E_{crash}^{after\_no\_treatment}$.

#### 3.2.2. Collision Diagram-Based Analysis

Collision diagrams are used to identify the patterns and locations where certain types of crashes occurred frequently. To develop a collision diagram, the first step was to draw the background map. After that, detailed police reports of crashes that occurred in this area were examined carefully. The locations of the crashes were marked on the map with different symbols representing different types of crashes.

**Figure 2:**
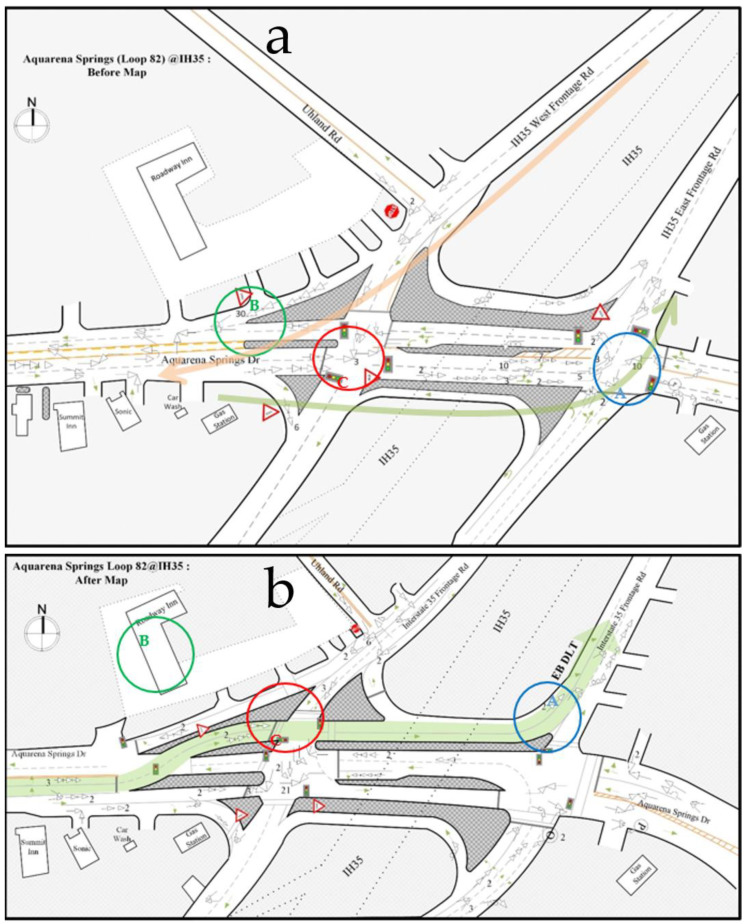
Collision diagrams at Intersection 1 (Loop 82, Aquarena Springs Drive at IH 35). (**a**) Collision diagrams before the implementation of DLT. (**b**) Collision diagrams after the implementation of DLT.

Figure 2 is one of the collision diagrams that we developed. In Figure 2, different symbols indicate different types of crashes, and the numbers beside the symbols are the numbers of the same type of crashes that occurred at the same location. There is a total of 11 different types of crashes marked in the collision diagrams, i.e., angle, dual left-turn angle, intersect, left-turn angle, rear-end, right-turn angle, head-on, U-turn, out of control, sideswipe, object, and pedestrian-related crashes. The definitions of these types of crashes along with their symbols are provided in Table 1.

The developed collision diagrams at both study locations before and after the implementation of DLT design are presented and discussed in the following sections.

## 4. Results

### 4.1. Intersection 1, Loop 82 (Aquarena Springs Drive) at IH 35

Intersection 1 is the first phase of the DLT intersection project in San Marcos, Texas. It is a signalized diamond interchange with two at-grade intersections at ramp terminals, where IH 35 frontage roads meet with Loop 82 (Aquarena Springs Drive). This location was converted to one DLT lane for the northbound traffic, which opened on 1 May 2014. The detailed before and after geometric conditions at this location are presented in Figure 2.

#### 4.1.1. Statistical Analysis

##### Direct Comparison between the before and after Crash Frequencies

**Figure 3:**
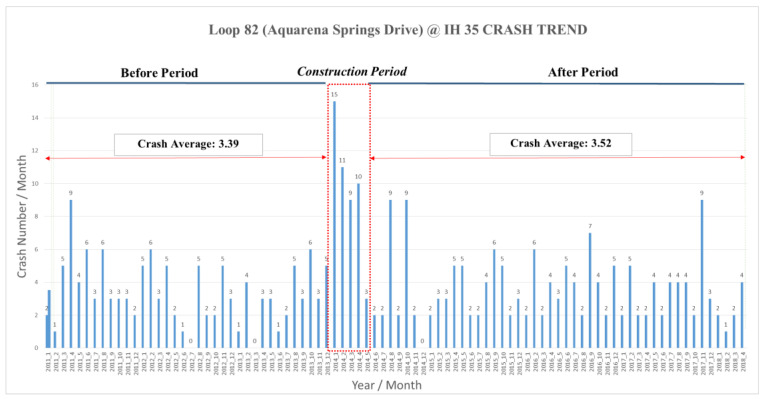
Monthly crash frequency during the study period (January 2011–April 2018) at Intersection 1 on Loop 82 (Aquarena Springs Drive) at (Interstate Highway) IH 35.

Figure 3 presents the monthly crash frequency during the study period. It can be seen that a relatively high number of crashes occurred during the construction period. The before-construction average crash frequency was 3.39 per month and, after the construction was completed, the average crash frequency was 3.52 per month. This suggested a slight increase of 4% in the crash frequency, but it was not a statistically significant difference because the *p*-value of the *t*-test statistic was 47% > 5%.

##### EB Method-Based before-and-after Comparison

As we mentioned before, to account for the effects of the changes in traffic volume before and after the implementation of DLT, the EB method was applied. According to Equation (2) and the traffic volume conditions at this location before and after the implementation of DLT, the expected crash frequency during the after-study time period under the assumption of no treatment, i.e., $E_{crash}^{after\_no\_treatment}$, was estimated and it was equal to 3.53/month (please see Table 2 for details). It was slightly higher than the actually observed crash frequency during the after-study time period, i.e., $E_{crash}^{after\_w\_treatment}=3.52/\mathrm{month}$. This result indicated that the implementation of DLT did not increase the crash frequency at this location.

#### 4.1.2. Collision Diagram-Based Analysis

Figure 2a presents the collision diagram for this intersection before the implementation of DLT. This diagram allowed the identification of the overall safety problems at this location, which are discussed as follows:

##### Safety Problems before the Implementation of DLT

Before the implementation of DLT at this location, there were two places where crashes occurred frequently (presented below) due to the following two heavy-traffic flows:

1. Traffic flow entering IH 35: Many vehicles move eastbound on Aquarena Springs Drive, make left turns at location A (blue circle), and then merge onto the IH 35 east frontage road. Due to the heavy left-turn traffic at location A, many crashes related to left turns occurred at this location, e.g., there were 10 left-turn angle crashes from January 2011 to December 2013. By conducting further analysis based on police crash reports, the following three major factors were identified that contributed to the high left-turn crash frequency before implementing DLT:
  (1) Disregarding the left-turn signage,
  (2) Drivers making left turns failed to yield the right of way, and
  (3) Drivers making dual left turns failed to keep their vehicles in a single lane.
2. Traffic flow exiting IH 35: Many vehicles exit from the southbound lanes of IH 35, merge onto the west frontage road of IH 35, and then make right turns at location B (green circle) to merge into the traffic on Aquarena Springs Drive. Before the implementation of DLT, this right-turn merge point was controlled by a “yield” sign. Due to the high volume of vehicles making right turns and the high volume of traffic on Aquarena Springs Drive, it is difficult for the right turn drivers to find a sufficient gap to make a safe merge, so a long queue of vehicles forms in the right turn lane, which is the reason for the high frequency of rear-end crashes at this location (30 crashes in 36 months).

##### Safety Problems after the Implementation of DLT

Figure 2b presents the collision diagram for this intersection after the implementation of DLT. By comparing the collision diagrams before and after the implementation of DLT, several findings related to the safety impacts of DLT were identified, and they are discussed below.

Finding A: Northbound left-turn-related crashes decreased after the implementation of DLT at location A (blue circle).

Since DLT eliminated left-turn movements at this intersection, only one left-turn crash had occurred here since DLT was implemented. This crash occurred in November 2014, a few months after the completion of the DLT project. The driver was unfamiliar with the new design and disregarded the “no left turn” sign late at night. Subsequently, no other left-turn crash had occurred at this location during the after-study period from May 2014 to April 2017.

Finding B: Rear-end crashes were reduced after the implementation of DLT at location B (green circle).

At this location, another safety benefit gained by implementing the DLT design was the reduction of rear-end crashes at the point where traffic from IH 35 westbound frontage road merges onto Aquarena Springs Drive (location B, green circle). After installing DLT, a traffic light was added at this point, and it coordinated with the signal light that was installed to control the DLT traffic at the left-turn crossover location. As a result of this change, the merging traffic can merge safely during the time the DLT traffic has the green light to move across to the left side. As a result, the frequency of rear-end crashes at this location decreased significantly to only 2 crashes in 35 months.

Finding C: Left-turn crashes increased after the implementation of DLT at location C (red circle).

Despite these improvements, some crash frequencies increased unexpectedly for the southbound left-turn traffic after the implementation of DLT. At location C (red circle) in Figure 2b, only one left-turn crash occurred before the implementation of DLT, while 21 left-turn crashes occurred at this location after the implementation of DLT.

Based on the descriptions in the police reports, the major cause for this type of crashes was that the left-turn drivers failed to yield the through traffic at the permissive left-turn phase. This was very likely caused by the geometric change after the implementation of DLT. To move the displaced left-turn traffic, an additional lane was added at the left-most side and the number of eastbound traffic through lane was reduced from two lanes to one lane. As a result, eastbound through traffic volume per lane increased, and it was difficult for the WB left-turn vehicles to find a safe space between the opposing through vehicles to make turns during the permissive phase.

### 4.2. Intersection 2, SH 80 (Hopkins Street) at IH 35

**Figure 4:**
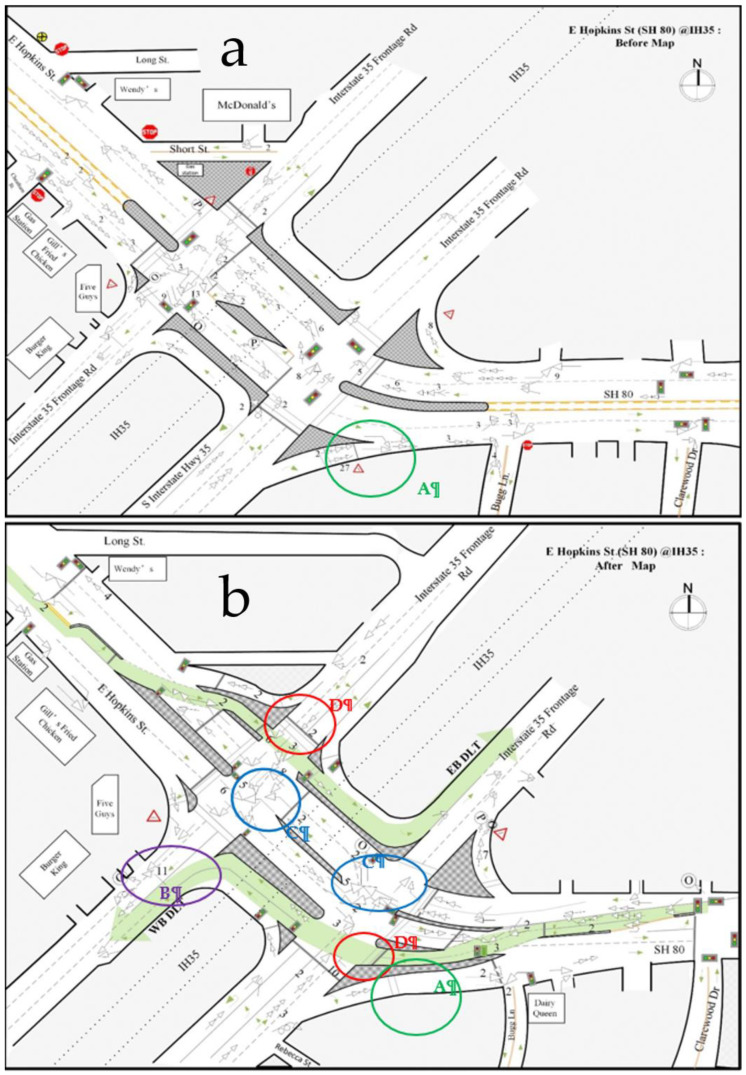
Collision diagrams at Intersection 2 SH 80 (Hopkins Street) at IH 35. Collision diagram-based analysis. (**a**) Collision diagrams before the implementation of DLT. (**b**) Collision diagrams after the implementation of DLT.

The second DLT intersection project on Hopkins Street opened on 12 October 2014, a few months later than the completion of the Aquarena Springs DLT project. It is a signalized diamond interchange with two at-grade intersections at ramp terminals, where IH 35 frontage roads meet with SH 80 (Hopkins Street). This location includes two displaced left-turn lanes, one of which allows eastbound traffic to turn north onto the IH35 frontage road (EB DLT), and the other allows the westbound traffic to move south of the IH 35 frontage road (WB DLT). The detailed before and after geometric conditions at this location are presented in Figure 4.

#### 4.2.1. Statistical Analysis

##### Direct Comparison between the before and after Crash Frequencies

**Figure 5:**
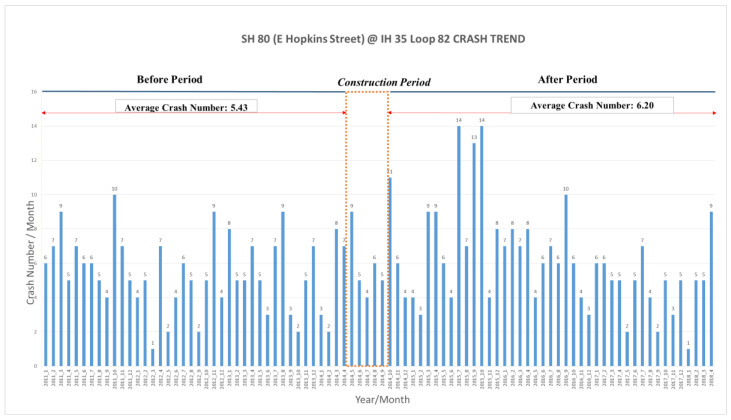
Monthly crash frequency during the study period (January 2011–12 October 2014) at Intersection 2 on State highway (SH) 80 (Hopkins Street) at IH 35.

Figure 5 presents the monthly crash data during the study period. Before DLT, the average crash frequency was 5.43 crashes per month; after DLT, the average crash frequency was 6.20 crashes per month. This was about a 14% increase in the crash frequency; however, it was also not a statistically significant increase (the *p*-value of the *t*-test statistic was 18% > 5%).

##### EB Method-Based before-and-after Comparison

The results of EB method-based before-and-after comparison are presented in Table 3. It can be seen that the traffic volume at this location increased by about 12.6%, which increased the estimated crash frequency during the after-study time period without any treatments, i.e., $E_{crash}^{after\_no\_treatment}$, to 7.11/month. It was about 15% higher than the observed crash frequency after the implementation of DLT during the same study time period, i.e., $E_{crash}^{after\_w\_treatment}$(6.20/month). This result indicated that the implementation of DLT actually reduced crash frequency by about 15% at this location.

Note that, at this study site, the two statistical methods employed by this research produced different results. Although the result of the direct comparison method indicated that the crash frequency increased by 14% after the implementation of DLT, the result of the EB method indicated that the implementation of DLT actually reduced crash frequency by about 15% by taking account of the effects of the increased traffic volume at this location. This result indicated that, when evaluating the effectiveness of a safety treatment, both the EB method and direct comparison method need to be used and their results need to be compared for a comprehensive evaluation.

#### 4.2.2. Collision Diagram-Based Analysis

##### Safety Problems before the Implementation of DLT

Figure 4a shows the collision diagram that was developed for this intersection before the implementation of DLT. The diagram indicated that the highest crash frequency was at location A (green circle), where the right turn traffic from the frontage road for IH35 south merges with the traffic on SH 80. Similar to Intersection 1, before the implementation of DLT, this right-turn merge point was controlled by a yield sign. Due to the high traffic volume on SH 80, it is difficult for right-turn vehicles to find sufficient gaps to merge safely, so a long queue forms in the right-turn lane and this caused the high frequency of rear-end crashes at this location (27 crashes in 43 months).

##### Safety Problems after the Implementation of DLT

Figure 4b presents the collision diagram for this intersection after the implementation of DLT. By comparing the collision diagrams before and after DLT, several findings related to the safety impacts of DLTs were identified and are discussed as follows:

Finding A: Rear-end crashes decreased after the implementation of DLT at location A (green circle).

After installing DLT, the rear-end crash frequency at location A was reduced significantly. Similar to Intersection 1, at this right-turn merge point, a traffic light was added, and the right-turn traffic can merge safely during the time the displaced left-turn traffic has the green light for moving across to the left side. As a result, the rear-end crash frequency at this location was reduced to only 1 crash in 43 months.

Finding B: Angle crashes increased at a downstream location of the WB DLT at location B (purple circle).

**Figure 6:**
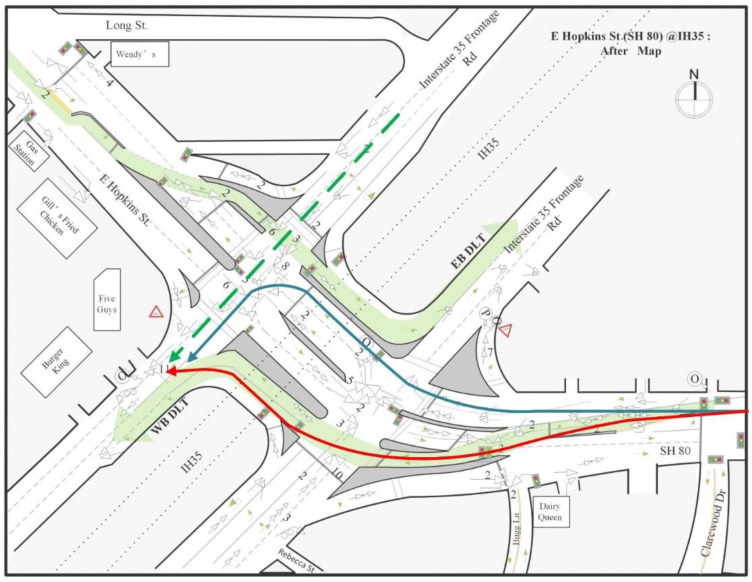
Comparison of the paths of southbound left-turn vehicles before and after the implementation of DLT.

As indicated by a purple circle in Figure 4b, 11 angle crashes occurred at a downstream location of the WB DLT near a driveway on the west frontage road for IH 35. The police crash reports for 11 crashes indicated that the high frequency of crashes involving right-turn angles occurred right after the displaced left-turn vehicles made left turns and tried to make right turns into the shopping plaza at the corner of this intersection. Figure 6 shows the vehicles’ travel paths before and after their left turns. The blue line represents the turning path before the implementation of DLT, and the red line represents the turning path after the implementation of DLT. The green dashed line represents the upstream through traffic from the frontage road for IH 35 west. Figure 6 shows that drivers of left-turn vehicles must make more lane changes to access the shopping plaza on the right side of the roadway because of the DLT design. These lane changes must be done immediately after they make turns through the DLT lane, which increases the chance of colliding with the through-movement vehicles from the frontage road of IH 35 west.

Finding C: Illegal left-turn crashes increased after the implementation of DLT at location Cs (blue circles).

At this intersection, there were 11 illegal left-turn crashes after the implementation of DLT because the drivers were unfamiliar with the design of this new intersection. Among the 11 crashes, 6 were southbound-turn crashes and 5 were northbound-turn crashes. All of these crashes occurred during the first year after the installation of DLT. According to police crash reports, most of these crashes occurred at night when there was inadequate lighting. Even though traffic signs prohibiting left turns were installed at these locations, drivers may still overlook or disregard them and make illegal left-turns, especially at night. Therefore, for the implementation of DLT intersections, improving the lighting conditions of intersections and installing more visible traffic signs (such as signs with flash beacons) are strongly recommended. In addition, more law enforcement and driver education are needed, especially during the first year after the implementation of DLT.

Finding D: T-bone angle crashes caused by running red lights increased after the implementation of DLT at location Ds (red circles).

From the crash diagram, we identified another type of crash that occurred frequently after the implementation of the DLT design, i.e., T-bone angle crashes between the DLT vehicles and the cross-street vehicles. Figure 4b shows that there were two hot spots of such crashes, i.e., location Ds, circled in red. The hot spot on the west side had six crashes and the hot spot on the east side had ten crashes. Both spots are close to the stop bars. The police crash reports indicated that all 16 of these crashes were caused by vehicles running red lights, mostly from the DLT approaches. This may be due to the fact that the drivers of the DLT vehicles did not expect that they need to stop at the main intersection; rather, they seem to have expected that they could move continually through the intersection after they got the green light to enter the DLT “channel”. This finding indicated that, in a DLT intersection, it is important to coordinate the signal timing between the left-turn crossover intersections and the main intersection to reduce the likelihood that DLT vehicles have to stop at the main intersection. In addition, it also was noticed that, at these locations, the stop bars of two conflicting approaches are very close. If drivers at one approach make a mistake and fail to stop their cars at the stop bar when the light turns red, there will not be enough time and space for the drivers at the conflict approach to make any evasive actions to avoid crashes. To prevent such T-bone crashes caused by running red lights, an advanced signal warning system could be used for both directions to ensure that drivers obey the traffic signals. In addition, driver education must be enhanced.

## 5. Conclusions and Recommendations

In this study, the safety impacts of the DLT design were assessed by conducting both statistical analysis and the analysis of collision diagrams based on the crash data and police crash reports collected over eight years at two intersections in San Marcos, Texas. These analyses were conducted before and after the implementation of DLT. It identified the following key findings:

(1) The implementation of DLT did not increase the overall crash frequencies at the studied intersections. It brought safety benefits at some locations for certain movements; meanwhile, it also caused safety problems at other locations for some other movements.
(2) In general, DLT has good performance in reducing left-turn-related collisions at the main intersections.
(3) DLT will also help right-turn vehicles merge safely and efficiently onto the intersection legs with DLT lanes, thereby reducing the rear-end crashes at the right-turn merge points.
(4) Many illegal left-turn crashes occurred at late night and during the first year after the implementation of DLT. Therefore, improving the lighting conditions of intersections and installing more visible traffic signs (such as signs with flash beacons) are strongly recommended. In addition, more law enforcement and driver education are needed, especially during the first year after the implementation of DLT.
(5) Left-turn vehicles are moved to the far-left side lane and, after making left turns from the DLT lanes, more lane changes are needed for them to access destinations on the right side of the roadway because of the design of DLT. The factors undoubtedly will increase the likelihood of collisions between the DLT vehicles and the through vehicles. Therefore, for DLT intersections where attractions are located (such as shopping plazas, gas stations, and hospitals), more access management is needed. For example, it is recommended that the entrance to the attractions be moved away from the area where the DLT exits are closely located to provide longer distances for DLT vehicles to make lane changes to access the attractions.
(6) At DLT intersections, it is important to coordinate the signal timing between the left-turn crossover intersections and the main intersection to reduce the likelihood that DLT vehicles will have to stop at the main intersection. In addition, advanced signal warning systems could be used to assist drivers in obeying the traffic signals.

The results of this study provide useful implications for both practitioners and researchers. First, in the implementation of DLT intersections, traffic engineers need to carefully consider different aspects of the intersection design, including access management, traffic signal coordination, and driver acceptance. Otherwise, DLT may mitigate the safety problem at some locations, but will cause more safety problems at other locations. Second, this study used both statistical analysis and collision diagram-based analysis. It was found that most of the key findings were obtained through the collision diagram-based approach. This result indicated that a collision diagram is an effective tool for analyzing the safety performance of a particular location such as an intersection. Currently, this method has not been widely used and most researchers are using the statistical analysis approach for crash risk analysis. Since the statistical analysis depends on the general crash attributes contained in the crash database, some local specific risk factors related to the crash location and its surrounding environment cannot be identified by the statistical analysis approach. Therefore, the collision diagram-based approach is recommended for intersection safety analysis because it can help us to better understand the safety problems at a particular intersection and identify effective solutions for these problems. Finally, given that these findings are based only on two selected DLT intersections in Texas, more studies at more DLT intersections are needed in the future to further validate and improve the findings of this study.

## Figures and Tables

**Table 1:** Symbols and definitions of types of crashes in the collision diagrams.

Type	Symbol	Definition
Angle	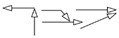	One through vehicle collides with another through vehicle in the crossing direction, or two vehicles collide while traveling in the same direction during lane change.
Dual left-turn angle	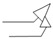	An accident involving two vehicles traveling in the same direction making a left turn while either driver fails to maintain her or his own lane and collides with the other vehicle.
Left-turn angle	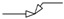	An accident involving two opposing vehicles when one of them is turning left.
Rear-end	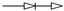	The front of one vehicle collides with the rear of another vehicle while both vehicles are traveling in the same direction.
Right-turn angle	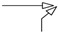	One right-turning vehicle collides with another vehicle on the cross street.
Head-on	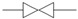	One vehicle hits another opposing vehicle’s front end.
U-turn	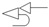	Crash occurs when one vehicle is performing a 180-degree turn to reverse the direction of travel and hits other vehicles.
Out of control	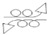	The vehicle starts to spin or twirl because of mechanical problems or driver-related effects, e.g., intoxication.
Sideswipe	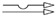	One vehicle impacts another vehicle traveling in the same direction by “swiping” along the surface in the direction of travel.
Object	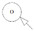	One vehicle hits a roadside object, e.g., the curb.
Pedestrian	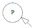	An accident in which a vehicle and a pedestrian collide.

**Table 2:** EB method-based before-and-after crash frequency comparison at Intersection 1 (Loop 82, Aquarena Springs Drive at IH 35).

$E_{crash}^{before}$ *	$AADT_{{}_{Ramp1}}^{before}$	$AADT_{{}_{crd1}}^{before}$	$AADT_{{}_{Ramp2}}^{before}$	$AADT_{{}_{crd2}}^{before}$	$VE_{before}^{}$	*b* **	$E_{crash}^{after\_no\_treatment}$ ***	$E_{crash}^{after\_w\_treatment}$ ****
3.39	12,807	29,761	17,787	11,214	580,612,545	0.9386	3.53	3.52
	$AADT_{{}_{Ramp1}}^{after}$	$AADT_{{}_{crd1}}^{after}$	$AADT_{{}_{Ramp2}}^{after}$	$AADT_{{}_{crd2}}^{after}$	$VE_{after}^{}$			
	15,759	25,962	20,077	9783	605,548,449			

* $E_{crash}^{before}$ was estimated based on the average crash frequency before the implementation of DLT. ** *b*, the coefficient of Volume Exposure (VE), was given by Wang et al. [20]. *** $E_{crash}^{after\_no\_treatment}$ was estimated according to Equation (2). **** $E_{crash}^{after\_w\_treatment}$ was estimated based on the average crash frequency after the implementation of DLT.

**Table 3:** EB method-based before-and-after crash frequency comparison at Intersection 1 (SH 80 (Hopkins Street) at IH 35).

$E_{crash}^{before}$ *	$AADT_{{}_{Ramp1}}^{before}$	$AADT_{{}_{crd1}}^{before}$	$AADT_{{}_{Ramp2}}^{before}$	$AADT_{{}_{crd2}}^{before}$	$VE_{before}^{}$	*b* **	$E_{crash}^{after\_no\_treatment}$ ***	$E_{crash}^{after\_w\_treatment}$ ****
5.43	17,787	21,330	39,430	29,714	1,551,019,730	0.9386	7.11	6.20
	$AADT_{{}_{Ramp1}}^{after}$	$AADT_{{}_{crd1}}^{after}$	$AADT_{{}_{Ramp2}}^{after}$	$AADT_{{}_{crd2}}^{after}$	$VE_{after}^{}$			
	20,095	23,784	43,499	36,539	2,067,349,441			

* $E_{crash}^{before}$ was estimated based on the average crash frequency before the implementation of DLT. ** *b*, the coefficient of VE, was given by Wang et al. [20]. *** $E_{crash}^{after\_no\_treatment}$ was estimated according to Equation (2) (# of crash per month). ****$E_{crash}^{after\_w\_treatment}$ was estimated based on the average crash frequency after the implementation of DLT.
